# The BioPAX Validator

**DOI:** 10.1093/bioinformatics/btt452

**Published:** 2013-08-05

**Authors:** Igor Rodchenkov, Emek Demir, Chris Sander, Gary D. Bader

**Affiliations:** ^1^The Donnelly Centre, University of Toronto, Toronto, Ontario M5S 3E1, Canada and ^2^Computational Biology Center, Memorial Sloan-Kettering Cancer Center, 1275 York Avenue, New York, NY, USA

## Abstract

**Summary:** BioPAX is a community-developed standard language for biological pathway data. A key functionality required for efficient BioPAX data exchange is *validation—*detecting errors and inconsistencies in BioPAX documents. The BioPAX Validator is a command-line tool, Java library and online web service for BioPAX that performs >100 classes of consistency checks.

**Availability and implementation:** The validator recognizes common syntactic errors and semantic inconsistencies and reports them in a customizable human readable format. It can also automatically fix some errors and normalize BioPAX data. Since its release, the validator has become a critical tool for the pathway informatics community, detecting thousands of errors and helping substantially increase the conformity and uniformity of BioPAX-formatted data. The BioPAX Validator is open source and released under LGPL v3 license. All sources, binaries and documentation can be found at sf.net/p/biopax, and the latest stable version of the web application is available at biopax.org/validator.

**Contact:** igor.rodchenkov@utoronto.ca or gary.bader@utoronto.ca

## 1 INTRODUCTION

BioPAX (Demir *et al.*, 2010) is a community-developed standard language for biological pathway data. BioPAX is defined in Web Ontology Language (OWL) and can represent a broad spectrum of biological processes including metabolic and signaling pathways, molecular interactions and gene networks. Pathguide.org (Bader *et al.*, 2006) lists the pathway databases and tools that support BioPAX.

Owing to the complexity of biological pathway knowledge and the rapid growth of the BioPAX corpus, manual validation and review of data exports is impractical. Unless this process is automated, a substantial number of errors would be left undetected and propagate to other resources and tools, making it more difficult for researchers to use pathway data, for each user must debug the errors, trace its origin and contact the data providers to fix them.

Other XML-encoded systems biology standards have developed custom validator services (Bornstein *et al.*, 2008; Czauderna *et al.*, 2010; Montecchi-Palazzi *et al.*, 2009). Although generic OWL validators can be used to perform basic BioPAX validation, they are not sufficient. The BioPAX specification contains a number of best practices and constraints that cannot be formally defined in OWL (e.g. regarding external ontology terms, standard identifiers or the constraint that each side of a transport interaction must contain participants from different cellular compartments). Checking some of these rules requires accessing external databases and non-OWL ontologies. Other constraints can only be checked algorithmically, such as circular nesting of protein complexes. Moreover, a tiered reporting format is required to differentiate between violations of invariants and best practices.

We developed a comprehensive BioPAX Validator that recognizes syntactic errors, semantic inconsistencies and violations of best practices in BioPAX models. These are defined as an expandable rule set written in Java and are reported in a human readable (HTML) format or XML. The validator can also normalize BioPAX models based on community-defined best practices and automatically fix some common errors. The validator substantially facilitates pathway data integration and analysis by increasing the conformity and uniformity of the available BioPAX-formatted data.

## 2 FEATURES

**Summary:** The validator can intercept and collect exceptions thrown by the BioPAX importer, the Paxtools (Demir *et al.*, 2013) StAX parser, such as a property range violation, unknown element (class) in the BioPAX namespace, unknown property, unclosed tag and other formatting and encoding issues, which are then reported as *range violated*, *unknown property*, *unknown class* and *syntax error*, respectively. Unlike other software capable of consuming BioPAX, the validator identifies many issues in one pass without failing, and thus reduces the number of iterations required to debug BioPAX document errors compared with typical BioPAX consumer software (like PaxTools) that fail fast. However, it is still recommended to fix major issues first and then repeat the validation, which will possibly identify additional issues.

**Expandable rule set:** Validation rules are defined as Java classes implementing a common interface, and new rules (Java classes) can be added. They can check aspects that involve individual BioPAX ontology elements, span multiple elements or the whole model and can use external resources. Rules can be configured to flag a warning or an error. There are currently >100 rules that are implemented based on the BioPAX model, specification, community discussions and best practices (biopax.org/validator/rules.html and the online documentation at biopax.org/m2site/). A Level1 or Level2 BioPAX model is automatically upgraded to Level3 before rules check it.

**Use cases:** The validator can be used as web application (online), locally from command line (batch mode) or integrated into third party software (library mode). The validator is accessible for online and/or non-Java applications as a web service. Batch mode is required for checking large BioPAX models, such as complete database exports, whereas library mode is typically used to check for correctness as a BioPAX model is being imported or manipulated by software tools.

The reference (standard) BioPAX Validator can be accessed as a web application via biopax.org/validator. Alternatively, and for batch checking file directories or large files (e.g. >100 Mb in total size), one can also download and run it as a console application locally. Users can also configure and deploy it on their own application server.

**Use of external vocabularies:** BioPAX uses a number of external controlled vocabularies, often defined as ontologies, (e.g. Gene Ontology cellular component subtree to specify the cellular location of physical entities). The validator currently uses seven OBO ontologies (as recommended in the BioPAX L3 specification): PSI-MI (molecular interactions), PSI-MOD (post-translational modifications), GO (gene ontology), CL (cell type), BTO (BRENDA tissue ontology), PATO (phenotypes) and SO (sequence ontology). To verify that terms from these ontologies are correctly used, the validator caches each controlled vocabulary locally for performance purposes. These caches can be automatically updated or configured to always use the latest web-based version.

**Normalization:** BioPAX allows significant syntactic freedom for data producers to facilitate data export. For example, data providers often assign non-standard local RDF identifiers (unique for each instance in a BioPAX document) for some utility classes. The normalizer can then replace private URIs of abstract BioPAX Level3 objects with standard Identifiers.org URIs as long as valid external references (UnificationXref) to standard databases, such as UniProt and NCBI taxonomy, are available in the file. Similarly, generic physical entities (e.g. the class of Wnt proteins) may be represented in multiple ways and these can be normalized to a standard form that is easier to use.

BioPAX normalization is not yet part of the formal BioPAX specification and guidelines; however, it is desired for data integration and required for semantic web and linked data use cases. The validator and normalizer bridge the gap between evolving BioPAX Level3 community best practices and multiple use cases from the community. Future work will involve extending the system to identify and fix additional errors, such as checking that the standard names of all human gene entities are valid HGNC gene symbols, all UniProt references are expressed in a standard manner and that post-translational modifications at specific protein positions are biologically valid (e.g. phosphorylation occurs at S, T or Y residues). Many of these features require access to large external databases.

**Tiered customizable reporting:** Rule violations are reported either in a human readable HTML format or XML. Three error levels are defined: *error* and *warning* for invariants and best practices, respectively, and *ignore* when a rule is not to be used. Users can configure rule behavior (i.e. ignore, warning, error) by defining a *profile*, and multiple profiles can be defined in a *configuration* file. Two pre-defined profiles are *default* and *notstrict*. Four error categories are used: *syntax, specification, recommendation and information*. Rules can report more than one *error code* (type). Other than the level, category and error code, the report involves an explanation of the error, common possible reasons that may lead to it and the list of cases with links (URIs) to the problematic model elements.

## 3 IMPLEMENTATION

BioPAX Validator is implemented in Java using the Spring framework and depends on the Paxtools API (Demir *et al.*, 2013) to read and manipulate BioPAX. We chose Java because some of the more complex rules (e.g. algorithmic) were easier to define in Java compared with other languages (e.g. OWL, rule-based languages).
